# Emerging Fluorescent Nanoparticles for Non-Invasive Bioimaging

**DOI:** 10.3390/molecules29235594

**Published:** 2024-11-26

**Authors:** Asma Khalid, Snjezana Tomljenovic-Hanic

**Affiliations:** 1 School of Physics, University of Melbourne, Parkville, VIC 3010, Australia; khalid@unimelb.edu.au; 2 School of Science, RMIT University, Melbourne, VIC 3001, Australia

**Keywords:** bioimaging, fluorescence, nanoparticles, biocompatibility, biodegradability

## Abstract

Fluorescence-based techniques have great potential in the field of bioimaging and could bring tremendous progress in microbiology and biomedicine. The most essential element in these techniques is fluorescent nanomaterials. The use of fluorescent nanoparticles as contrast agents for bioimaging is a large topic to cover. The purpose of this mini-review is to give the reader an overview of biocompatible and biodegradable fluorescent nanoparticles that are emerging nanomaterials for use in fluorescent bioimaging. In addition to the biocompatibility of these nanomaterials, biodegradability is considered a necessity for short-term sustainable bioimaging. Firstly, the main requirements for bioimaging are raised, and a few existing fluorescent nanoprobes are discussed. Secondly, a few inert biocompatible fluorescent nanomaterials for long-term bioimaging that have been, to some extent, demonstrated as fluorescent probes are reviewed. Finally, a few biocompatible and biodegradable nanomaterials for short-term bioimaging that are evolving for bioimaging applications are discussed. Together, these advancements signal a transformative leap toward sustainability and functionality in biomedical imaging.

## 1. Introduction

In fluorescence-based techniques, fluorescent fluorophores represent the most important element of fluorescent spectroscopy. However, in a biological cell, the presence of components such as collagens and flavins produces fluorescent background signals. These components typically absorb light in the range of 300–500 nm and fluoresce at 400–550 nm [1]. Therefore, it is essential for the fluorescent probe to absorb light at wavelengths longer than 500 nm and emit light at wavelengths longer than 600 nm. Fluorescent probes should also display a high fluorescence quantum yield and large Stokes shift. For in vivo applications, the main criteria for optical imaging techniques are biocompatibility, photostability, suitable wavelengths of absorbance, and fluorescence that differs from tissue auto-fluorescence. Additionally, the light emission should be in the near-infrared region (NIR), 700–900 nm, as it penetrates centimeters into the tissue, whereas visible light can only travel microns [2]. Within this field, imaging with NIR light not only gives penetration depth but also increases the signal-to-background ratio when using contrast agents.

Organic dyes are among the most popular fluorescent probes due to their low cost and small molecular size [3]. They emit light in broad-spectrum regions ranging from UV to the far-infrared region, depending on their chemical structure [4]. However, organic dyes that are used as contrast agents for NIR imaging are inherently toxic and suffer from poor hydrophilicity and photostability [5]. Additionally, they exhibit low quantum yields with low detection sensitivity and have insufficient stability in biological systems [6]. In order to improve imaging efficiency, these dyes have been conjugated with other nanoparticles (NPs) and biological macromolecules through chemical conjugation methods that, in turn, may compromise the imaging properties [7]. There are also fluorescent proteins (green fluorescent proteins, monomeric teal fluorescent proteins, etc.) that vary in color and are currently used as tags for bioimaging applications [8]. Most of these proteins are excited in the UV region, which interferes with the intrinsic fluorescence of live cells, and complete information about the structure may not be revealed.

**Figure 1:**
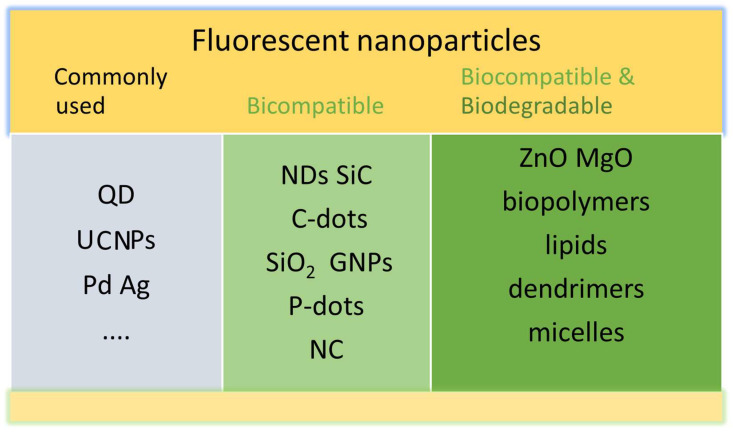
Some nanoparticles used in bioimaging. QD: quantum dots, UCNPs: up-conversion NPs, Pd: palladium, Ag: silver, ND: nanodiamond, SiC: silicon carbide, C-dots: carbon dots, SiO_2_: silica, GNPs: gold NPs, P-dots: polymer dots, NC: nanocellulose, ZnO: zinc oxide, MgO: magnesium oxide. In this review, fluorescent NPs are broadly classified into two main categories of importance for bioimaging: biocompatible and (ii) biocompatible and biodegradable NPs.

To overcome the limitations of organic dyes and fluorescent proteins, various NPs, including quantum dots, noble metal clusters (Au NP, Pd NPs, Ag NPs), and carbon-based nanomaterials (carbon dots, graphene, nanodiamonds, etc.) have been developed and widely studied in biological imaging [9]. Some of these NPs are classified in Figure 1 in terms of their biocompatibility and biodegradability.

Quantum dots (QDs) have been investigated due to their brightness [10], but the major obstacle in their clinical use remains their toxicity [1]. Individual Ag NPs likewise have been reported to emit photoluminescence in the visible-to-NIR range when excited with a blue laser. However, they exhibited fluorescence intermittency and spectral fluctuation under continuous laser excitation [11]. Additionally, pump wavelengths below 500 nm are known to activate autofluorescence from intracellular components, which limits the suitability of Ag NPs for bioimaging applications.

In this review, fluorescent NPs are broadly classified into two main categories of importance for bioimaging: (i) biocompatible and chemically inert NPs for long-term bioimaging and (ii) biocompatible and biodegradable NPs for short-term bioimaging. Toxicity of NPs is a growing concern in biomedicine [12]. In particular, NPs of less than 100 nm that are of similar dimensions as biomolecules such as DNA, enzymes, and proteins present in living cells may pose potential consequences on human health [13]. Various in vitro and in vivo evaluation methods of biocompatibility of nanomaterials have been reported, with sometimes conflicting findings regarding NP biocompatibility. It is hard to compare those reports as a wide range of different cell lines and NP size, shape, and concentrations were used in those studies. Although it is possible to encapsulate many NPs with biocompatible nanomaterials, in this review, we consider only inherently biocompatible nanomaterials (Section 2). In addition to biocompatibility, real-time short-term bioimaging prefers candidates exhibiting biodegradable properties. Biodegradable nanomaterials can be naturally degraded under biological conditions in the body [14,15]. While each biodegradable nanomaterial has its advantages and disadvantages, biodegradable NPs hold great future potential in short-term bioimaging.

## 2. Chemically Inert Biocompatible Nanoparticles for Long-Term Bioimaging

In long-term bioimaging applications, particularly for continuous monitoring and diagnostic purposes, chemically inert biocompatible NPs play a critical role due to their exceptional stability and resistance to degradation within biological systems. These NPs are ideal for imaging scenarios that require prolonged stability and minimal interaction with their environment, ensuring that the particles remain photostable and do not interfere with cellular processes over time. Such properties make chemically inert NPs essential for detailed cellular imaging, tissue tracking, and even in vivo applications where persistent, high-contrast imaging is necessary. Unlike biodegradable NPs designed for short-term usage, chemically inert NPs offer prolonged bioavailability and a consistent signal, which is crucial for applications such as tumor monitoring, which demands long-term imaging without the need for repeated administrations. Given the stability and biocompatibility of materials like nanodiamonds, gold, and silicon carbide, these NPs meet the stringent criteria for in vivo imaging, offering the possibility for non-invasive, continuous monitoring of dynamic biological processes without compromising cell health or function.

### 2.1. Nanodiamonds

Nanodiamonds (NDs) possess significant potential in fluorescence bioimaging owing to their unique properties, which include exceptional hardness, high Young’s modulus, biocompatibility, broad energy band gap, and thermal conductivity (2 × 10^3^ W/m·K) [16]. These attributes, inherited from bulk diamond, make NDs highly stable under extreme thermal, chemical, and mechanical conditions. Importantly, NDs bring these macroscopic properties to the nanoscale, opening a range of applications, particularly in biomedical research.

First produced over fifty years ago through detonation methods [17], NDs are formed by controlled explosive processes, producing soot containing 4–5 nm diameter particles that cluster into larger aggregates (100–200 nm) [18]. Other ND synthesis methods include laser ablation, high-energy ball milling [19], and plasma-assisted chemical vapor deposition (CVD) [20]. Despite the small size of detonation NDs (usually less than 10 nm), they tend to agglomerate, forming micron-sized aggregates due to surface interactions and bonding [21]. However, recent advances in wet-stirred media milling have enabled the preparation of single-digit NDs in colloidal suspension [22]. This method breaks down aggregates, dispersing NDs effectively in water, thus enhancing their applicability in biomedical settings.

**Figure 2:**
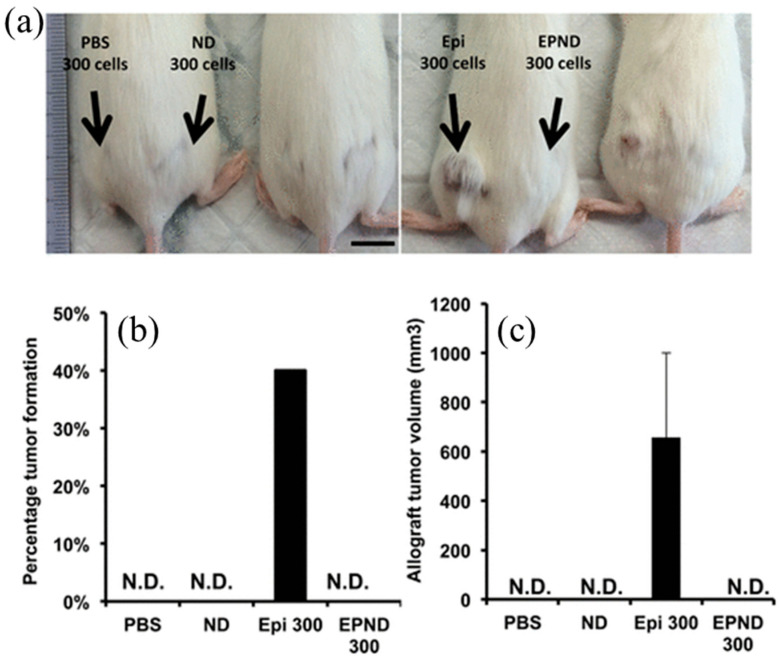
Representative images showing the uptake of epirubicin-loaded nanodiamonds (NDs) in chemoresistant hepatic cancer stem cells (HCSCs). Panel (**a**) displays the bright-field image of HCSCs, highlighting cell morphology. Panel (**b**) illustrates the fluorescence distribution of epirubicin-loaded NDs within the cells, confirming intracellular uptake. Panel (**c**) presents the merged image of panels (**a**,**b**), providing a comprehensive view of ND localization within HCSCs. Scale bar: 10 μm. Adapted from Wang et al. (2014) [23].

A recent study (Figure 2) [23] illustrates the use of epirubicin-loaded NDs (EPND) for therapeutic applications, specifically in inhibiting tumor initiation in a murine hepatic tumor allograft model. Figure 2a presents representative images of mice injected with cells derived from PBS, nanodiamond, epirubicin, or EPND-treated MYC-driven tumors. The results highlight that mice receiving EPND-treated tumor cells showed no detectable tumor formation, in stark contrast to significant tumor growth in other groups. Figure 2b provides a quantitative analysis of tumor formation rates, demonstrating a substantial reduction in allograft tumor initiation with EPND treatment. This figure underscores the efficacy of NDs as a drug delivery platform, particularly in overcoming chemoresistance in cancer stem cells and inhibiting tumor recurrence. These findings exemplify the potential of NDs to integrate imaging and therapeutic capabilities, paving the way for advanced theranostic applications.

The inherent chemical inertness and low toxicity of NDs have made them attractive for cellular imaging as well as drug delivery and applications. Their surfaces can be modified with various functional groups for targeted delivery, enhancing the interaction with bioactive molecules [24]. The structure of NDs follows a core-shell model, with an sp^3^-bonded diamond core often coated by graphitic or amorphous carbon. This coating may carry functional groups such as carboxyl, hydroxyl, or ketone [25]. Surface modification is essential, as it enables the attachment of biomolecules, drugs, or proteins to NDs, facilitating specific interactions within biological systems. Modifications can be achieved through oxidation or chemical treatments, creating a controlled environment suitable for bioconjugation [26].

NDs’ biocompatibility has been confirmed through various in vitro and in vivo studies, including microinjection experiments in Caenorhabditis elegans, which showed no adverse effects on the organisms’ reproductive capabilities or survival [27]. Nevertheless, the surface chemistry of NDs significantly impacts their cytotoxicity, highlighting the importance of testing modified NDs before their use in biomedical applications [28]. For example, studies have demonstrated higher cytotoxicity in amine-terminated NDs compared to hydroxyl- or carboxyl-terminated ones [28]. Therefore, it is critical to ensure that NDs intended for use in biological environments maintain non-toxic properties.

ND aggregation remains a challenge, as their small size and high surface energy lead to the formation of aggregates. Methods such as media milling and centrifugation have been developed to address this issue, improving dispersion stability [29]. Coating NDs with polymers or silica has proven effective in preventing re-aggregation, enhancing colloidal stability, and providing platforms for further functionalization [30,31].

The fluorescence in NDs primarily originates from structural defects within their diamond lattice. These defects, known as color centers, include a variety of configurations like silicon-vacancy (SiV) and nitrogen-vacancy (NV) centers, each emitting light at specific wavelengths [16,20]. The NV center, particularly in its negatively charged state (NV⁻), is the most extensively studied due to its photostability and emission properties in the visible to NIR [32]. NV centers emit light at 637 nm with a broad sideband extending to around 850 nm, making them highly suitable for bioimaging due to minimal tissue autofluorescence interference [33].

NV⁻ centers in NDs demonstrate high quantum efficiency (0.7–1.0) and photostability, essential for long-term imaging applications. Studies have shown that these centers exhibit no photoblinking or photobleaching, even under continuous excitation, which distinguishes them from traditional fluorophores like quantum dots or organic dyes [24]. This makes them ideal for prolonged imaging sessions, allowing for real-time tracking of cellular and molecular processes. Wide-field fluorescence microscopy revealed that these NDs, due to their photostable properties and NV center emissions, could be tracked effectively inside the cells for extended periods without signs of photobleaching or cytotoxic effects [34].

In biomedical research, NV centers within NDs have been successfully used as cellular markers and imaging agents due to their biocompatibility and stable emission properties. Functionalization further enhances their uptake and distribution within cells, enabling targeted imaging applications such as drug delivery or tracking cellular dynamics [19,35]. The brightness of NDs can be tailored by increasing the nitrogen content and optimizing the conditions for NV center formation [33].

Hence, the versatile surface chemistry, biocompatibility, and stable fluorescence of NDs, particularly those incorporating NV centers, present promising avenues for both in vitro and in vivo imaging applications. These features enable precise, high-resolution imaging while offering the potential for integration into theranostic platforms, where diagnostics and therapeutic delivery converge. Continued advancements in ND fabrication and functionalization are expected to further solidify their role in bioimaging, enhancing both the scope and efficacy of biomedical applications.

### 2.2. Gold Nanoparticles

Gold NPs (GNPs) are widely researched for their applications in bioimaging, owing to their unique optical properties, biocompatibility, and ease of functionalization. Gold’s inert nature, combined with its capacity to absorb and scatter light strongly at certain wavelengths, provides a basis for developing highly sensitive and specific imaging probes. GNPs exhibit localized surface plasmon resonance (LSPR), a phenomenon where free electrons on the particle surface oscillate in response to incident light, leading to enhanced scattering and absorption. This LSPR effect can be precisely tuned by adjusting the size, shape, and environment of the NPs, making GNPs versatile tools for various imaging modalities [35].

**Figure 3:**
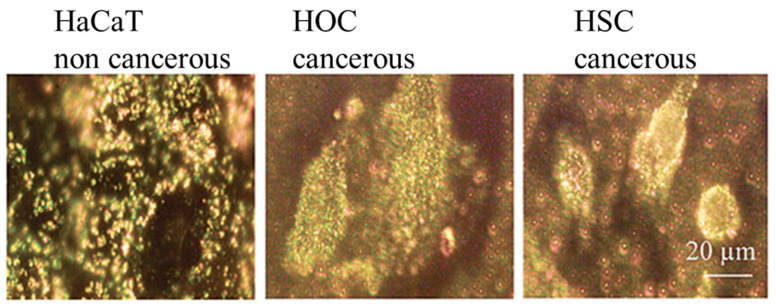
Surface plasmon resonance (SPR) scattering images and absorption spectra of HaCaT noncancerous cells (**left column**), HOC cancerous cells (**middle column**), and HSC cancerous cells (**right column**) after incubation with anti-EGFR antibody-conjugated gold NPs. SPR scattering highlights distinct binding patterns, with GNPs accumulating specifically on the surface of cancer cells due to overexpressed EGFR, creating sharp spectral peaks near 545 nm. Noncancerous cells exhibit less specific binding, resulting in broader spectra. These results demonstrate the potential of GNPs for distinguishing cancerous and noncancerous cells. Image reprinted from [37].

In fluorescence imaging, GNPs can be used either as direct fluorescence enhancers or as quenchers when conjugated with fluorophores. The proximity between GNPs and fluorophores plays a critical role, as the NPs can either enhance or quench fluorescence depending on the distance. This tunable interaction allows for the development of molecular probes capable of detecting specific biomolecules with high sensitivity and precision [36]. The surface plasmon resonance scattering and absorption of anti-EGFR antibody conjugated gold NPs have shown strong potential for cancer cell imaging and diagnostics [37], as illustrated in Figure 3. Furthermore, GNPs conjugated with antibodies or aptamers have been employed to target and visualize specific proteins or cellular components, increasing the specificity and sensitivity of fluorescence-based bioimaging techniques [38].

Beyond fluorescence imaging, GNPs are also highly effective in dark-field microscopy due to their strong scattering properties. When exposed to light, the LSPR effect causes GNPs to scatter light at specific wavelengths, producing a bright contrast against dark backgrounds, even when the NPs are at low concentrations. This characteristic has been particularly useful in detecting single cells or molecules, making dark-field microscopy a powerful technique for real-time tracking and imaging of cellular processes [39].

For in vivo imaging, GNPs offer advantages in photoacoustic imaging (PAI) and computed tomography (CT). In PAI, GNPs convert absorbed light into heat, generating ultrasonic waves that can be detected to form high-resolution images of tissues. GNPs can be functionalized to target specific tissues or tumors, enhancing the accuracy and depth of imaging. This method has shown promise for detecting early-stage cancers and monitoring therapeutic responses [40]. In CT imaging, the high atomic number of gold allows GNPs to serve as effective contrast agents, providing superior imaging contrast compared to conventional agents like iodine. GNPs accumulate preferentially in tumors due to the enhanced permeability and retention (EPR) effect, thus enabling detailed imaging of tumor vasculature and morphology [41].

One of the main advantages of GNPs in bioimaging is their biocompatibility. Gold is generally considered non-toxic, and when properly coated with biocompatible polymers like polyethylene glycol (PEG), GNPs can circulate in the bloodstream for extended periods without provoking an immune response [42]. However, the size, shape, and surface charge of GNPs significantly influence their biocompatibility and biodistribution, making surface modification crucial for clinical applications. Studies have shown that PEGylation, for instance, enhances the circulation time and reduces the clearance rate of GNPs by the reticuloendothelial system (RES), making them more effective for in vivo imaging and drug delivery [43].

Another application is the use of GNPs as theragnostic agents—combining diagnostic imaging with therapy. For example, GNPs have been used in photothermal therapy (PTT), where they absorb NIR light and convert it into heat, effectively killing targeted cancer cells. This dual capability not only allows visualization of the tumor site but also offers a means of targeted treatment, thereby enhancing the efficacy of cancer therapies [44].

Hence, the versatility of GNPs in imaging techniques—ranging from fluorescence and dark-field microscopy to CT and photoacoustic imaging—highlights their potential to advance both in vitro and in vivo imaging capabilities. Through appropriate surface modifications, GNPs can be tailored to target specific cells or tissues, providing precise and high-contrast imaging while maintaining biocompatibility. As research progresses, further exploration of GNPs in combination with therapeutic agents may lead to novel theragnostic applications that merge diagnosis and treatment into a single platform.

### 2.3. Silicon Carbide Nanoparticles

Silicon carbide nanoparticles (SiC NPs) have gained attention in the biomedical imaging field due to their unique physical and chemical properties. SiC, a wide-bandgap semiconductor, is known for its exceptional mechanical strength, thermal conductivity, and chemical stability, which make it a robust platform for biological applications, including bioimaging and sensing [45]. The intrinsic properties of SiC NPs, such as biocompatibility and resistance to oxidation, allow for their use in both in vitro and in vivo environments without significant degradation, making them an attractive alternative to other traditional nanomaterials [46].

**Figure 4:**
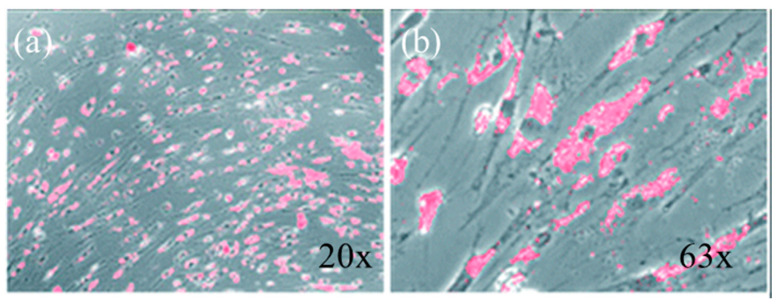
Bright-field and fluorescence overlay image of mesenchymal stem cells labeled with SiC NPs after 11 days of incubation at (**a**) 20× and (**b**) 63× magnifications. The persistent fluorescence signal highlights the stability and retention of SiC NPs within the cellular environment, demonstrating their suitability for long-term imaging applications in biological systems. Image reprinted from [50].

One of the primary advantages of SiC NPs in bioimaging is their ability to emit fluorescence in the visible and NIR range when doped or functionalized with specific elements like nitrogen or rare earth ions [47]. The tunable photoluminescence of SiC NPs enables their application in multicolor imaging and tracking at cellular and molecular levels. Studies have demonstrated that SiC NPs exhibit photostable fluorescence, allowing long-term imaging without photobleaching, a common issue observed with traditional organic dyes and even some other fluorescent nanomaterials like quantum dots [48,49]. This photostability is particularly valuable for real-time monitoring of biological processes. An example is shown in Figure 4, where SiC NPs have been demonstrated as a photoacoustic and photoluminescent dual-imaging contrast agent for long-term cell tracking. The excitation wavelength used is 405 nm, while the emission wavelength range for the SiC NPs is recorded from 450 nm to 550 nm, peaking around 500 nm.

The biocompatibility of SiC NPs has been extensively tested, with multiple studies confirming low cytotoxicity levels across different cell lines, including HeLa and fibroblast cells [51]. The surface chemistry of SiC can be easily modified to enhance biocompatibility further or to attach targeting ligands for specific cell labeling [52]. Functionalization with biomolecules such as antibodies and peptides has enabled the targeted delivery of SiC NPs to cancer cells, providing a pathway for precision imaging and diagnostics [53]. Additionally, the surface of SiC NPs can be tailored with hydrophilic coatings, improving their dispersion in aqueous solutions, which is essential for biomedical applications [54].

In vivo studies highlight the promise of SiC NPs for imaging applications. Their ability to penetrate tissues with minimal toxicity makes them suitable for whole-body imaging in animal models [47]. Recent advances have shown that SiC NPs, particularly those doped with nitrogen, can be employed for deep-tissue imaging due to their emission in the NIR, which experiences less scattering and absorption by biological tissues [48]. This feature facilitates the monitoring of physiological changes within organs and tissues, such as inflammation or tumor progression [55]

However, while SiC NPs show potential, there are challenges associated with their use. For example, the synthesis of SiC NPs with consistent size and shape remains technically demanding, and variations in particle size can influence their optical properties and biological behavior [56]. Moreover, ensuring stability and minimizing aggregation in biological environments remain critical issues, as SiC NPs tend to agglomerate, reducing their effectiveness in vivo [57]. Addressing these concerns requires further advancements in surface modification techniques to maintain stability and biocompatibility under physiological conditions [58].

Overall, SiC NPs represent a promising tool for bioimaging due to their photostability, biocompatibility, and ability to be functionalized for targeted applications. Continued research and development in their synthesis and surface modification will be crucial for fully realizing their potential in clinical and diagnostic settings.

### 2.4. Nanocellulose

Nanocellulose (NC) is a versatile biomaterial derived from natural cellulose sources, including plants, bacteria, and algae. Its nanoscale forms, like cellulose nanofibers (CNFs) and cellulose nanocrystals (CNCs), exhibit remarkable properties such as high mechanical strength, biocompatibility, and chemical versatility, making it a promising candidate for a wide range of biomedical applications [59,60]. These include drug delivery systems, tissue engineering scaffolds, and components in wound dressings [61].

**Figure 5:**
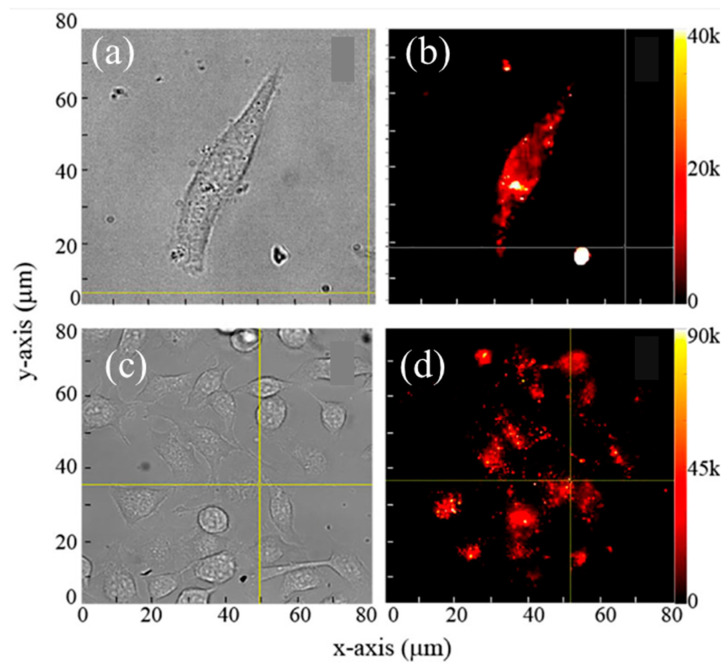
(**a**) Bright-field microscopic and (**b**) fluorescence microscopy images showing cellulose nanoparticles (CNPs) localized within human skin keratinocyte cells. (**c**) Bright-field and (**d**) fluorescence microscopy images illustrating the internalization of cellulose nanofibers (CNFs) within skin cells. These images demonstrate the photostable intrinsic fluorescence of cellulose-based materials, enabling their potential use in non-invasive cellular imaging. Image reprinted from [61].

Nanocellulose’s intrinsic fluorescence has recently been explored, particularly in the context of bioimaging [61]. This fluorescence, which is predominantly in the red spectral range, offers an advantage for bioimaging applications because it minimizes interference with cellular autofluorescence, which typically occurs in the blue and green regions of the spectrum [61]. The red emission wavelengths (>600 nm) of NC materials are well-suited for in vitro imaging, allowing clear visualization of cellular structures without the need for additional labeling or dyes, as shown in Figure 5. This intrinsic fluorescence, combined with NC’s biocompatibility, enables non-invasive imaging that does not alter the biological environment, offering a powerful tool for cellular studies.

In vitro studies have shown that nanocellulose, when integrated into human cells like keratinocytes and cancer cells, exhibits stable and bright fluorescence, facilitating the monitoring of cellular uptake and internalization processes. Confocal imaging experiments demonstrated that NC fibers and NPs remain photostable within cells, allowing extended observation times without significant photobleaching or blinking. This photostability, coupled with the ability of NC to emit in the NIR, makes it particularly advantageous for long-term cell tracking and in-depth imaging studies [61].

One of the key benefits of using nanocellulose in bioimaging is its low cytotoxicity, which is critical for applications involving live cells. Cytotoxicity tests reveal that NC does not significantly affect cell viability even at higher concentrations, affirming its biocompatibility [62]. Furthermore, its capacity for surface functionalization opens up the possibility of conjugating NC with bioactive molecules, enhancing its potential as a multifunctional platform for targeted imaging and therapeutic delivery. The ability to attach functional groups like carboxyl or hydroxyl groups to the NC surface allows it to be tailored for specific applications, such as delivering fluorescent markers or therapeutic agents directly to target cells [61].

However, while NC shows promise, challenges remain. One notable limitation is its variability in toxicity, which depends on the particle size and surface chemistry. Smaller CNCs and CNFs, while useful for penetrating cellular structures, might present greater cytotoxic risks if their surface is not adequately modified or purified [62]. Additionally, nanocellulose fibers tend to aggregate, which could limit their dispersibility and stability in biological environments. Further research into stabilizing agents and surface modifications is necessary to ensure consistent behavior and performance in bioimaging applications.

Hence, nanocellulose’s intrinsic fluorescence and biocompatibility make it a valuable tool for bioimaging, particularly for in vitro cell studies. Its potential to provide non-invasive, long-term imaging without the need for additional chemical labels sets it apart from other fluorescent NPs. However, optimizing its surface chemistry and addressing aggregation issues remain crucial steps for its broader application in biomedical fields.

### 2.5. Polymer Dots

**Figure 6:**
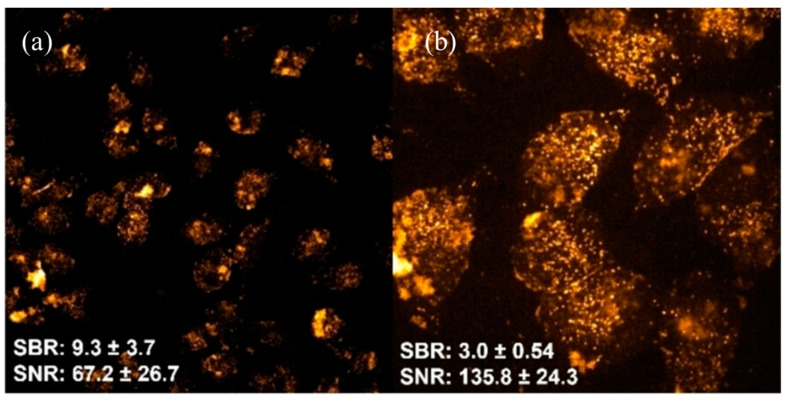
Confocal fluorescence images of fixed HeLa cells incubated with polymer dots (P-dots) functionalized with a guanidine-mimic monomer (Pdot-BGN-B) at a concentration of 12.5 μg/mL for 6 h at (**a**) 20× and (**b**) 63×. Values of signal-to-noise ratio (SNR) and signal-to-background ratio (SBR) were calculated based on the average fluorescence intensity from 45 cells imaged with a (**a**) 20× and (**b**) 63× objective lens. Fluorescence was excited at 488 nm, and emission was recorded within the 570–620 nm range, showcasing the efficient cellular uptake and bright fluorescence of the P-dots. Image reprinted from [64].

Polymer dots (P-dots) are gaining recognition as advanced materials in fluorescence bioimaging due to their excellent photophysical characteristics, including high fluorescence brightness, large absorption cross-sections, and exceptional resistance to photobleaching. These dots are primarily composed of π-conjugated polymers such as polyfluorene, poly(p-phenylene vinylene) (PPV), and poly(thiophene). The π-conjugated structure allows P-dots to exhibit intense fluorescence and broad absorption spectra, making them versatile for various biological applications, including both in vitro (Figure 6) and in vivo imaging [63].

P-dots are typically synthesized via nanoprecipitation, a method that involves dissolving the polymer in a water-miscible organic solvent followed by its rapid mixing with an aqueous solution. This process results in the formation of nanoscale P-dots with a hydrophobic polymer core surrounded by hydrophilic functional groups that stabilize the particles in water [65,66]. The size and surface properties of P-dots can be controlled during this process, allowing the fabrication of particles suited for specific imaging needs.

The application of P-dots in fluorescence bioimaging is enhanced by their ability to be functionalized with various biomolecules. The surface of P-dots can be modified with antibodies, peptides, or nucleic acids, enabling them to target specific cellular markers or biological processes [67]. This functionalization makes P-dots highly effective for cell labeling and real-time monitoring of cellular events. Moreover, their bright and stable fluorescence provides clear imaging results even over extended observation periods, surpassing traditional fluorescent dyes and other NPs like quantum dots [67].

In vivo imaging benefits from P-dots’ ability to emit fluorescence in the NIR, which is particularly advantageous for deep tissue imaging. NIR emission minimizes background autofluorescence from biological tissues, allowing P-dots to penetrate deeper into tissues and provide high-contrast imaging of structures such as tumors and blood vessels [68]. This feature makes P-dots suitable for monitoring therapeutic responses and tracking drug delivery pathways in animal models.

Despite these advantages, there are challenges associated with P-dots, such as achieving uniformity in size and fluorescence properties across different batches. Additionally, their long-term biocompatibility, especially in clinical applications, remains a critical area for further study. Understanding and minimizing any potential toxicity of P-dots is necessary to advance their use in clinical diagnostics and therapeutics [67].

P-dots hold great promise for the future of bioimaging. Their tunable properties, high brightness, and ability to be modified for targeting specific cellular components make them valuable tools for developing advanced imaging techniques in biology and medicine.

### 2.6. Carbon Dots

Carbon dots (CDs) are a class of carbon-based NPs, generally smaller than 10 nm, known for their exceptional biocompatibility and versatile fluorescence properties. CDs can be synthesized from a variety of carbon-rich sources, including organic molecules (e.g., citric acid), biomass (e.g., orange peels), and carbon-based materials like graphene or carbon nanotubes. These carbon sources undergo different fabrication methods, such as laser ablation, electrochemical oxidation, and hydrothermal carbonization, yielding particles with tunable photoluminescence characteristics depending on synthesis conditions [69,70]. The small size and surface functional groups of CDs allow for the modulation of their optical properties and enable their widespread use in bioimaging.

The fluorescence of CDs arises due to quantum confinement and the presence of surface defects, which can be influenced by surface chemistry, particle size, and doping with heteroatoms like nitrogen and sulfur. Doping is particularly effective in enhancing fluorescence intensity and tuning the emission spectrum of CDs, making them versatile for bioimaging applications. This flexibility allows CDs to emit light across a broad spectrum, including the visible and NIR, which is critical for deep-tissue imaging applications. NIR-emitting CDs are especially advantageous in vivo, as these wavelengths minimize interference from biological autofluorescence and enable deeper tissue penetration [71,72].

In vitro applications of CDs demonstrate their ability to penetrate cellular membranes and localize within specific cellular compartments, such as the cytoplasm, providing bright, stable fluorescence for high-resolution imaging. The use of surface functionalization techniques, including PEGylation, has further enhanced the biocompatibility and targeting capabilities of CDs, allowing them to be modified with antibodies, peptides, or other molecules for precise cellular imaging [73]. CDs are often used to label specific proteins or cellular organelles, offering a powerful tool for studying cellular processes and interactions in real time [74].

**Figure 7:**
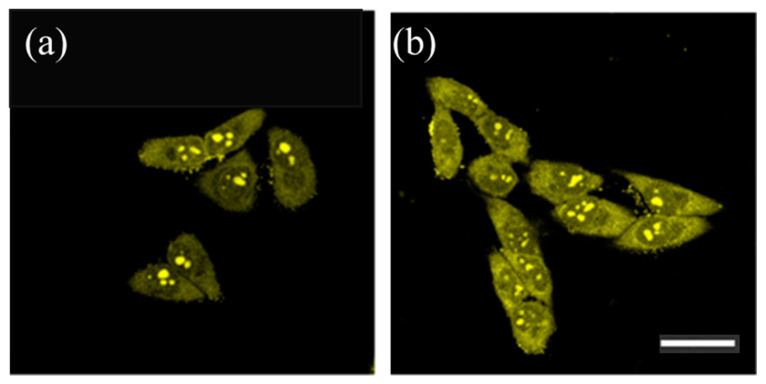
Confocal fluorescence images of living HeLa cells incubated with carbon dots (5 μg/mL) after (**a**) 90 s and (**b**) 5 min of incubation. The excitation wavelength was set at 488 nm, and the emission range was recorded between 500 and 600 nm. These images highlight the carbon dots’ suitability for cellular imaging, demonstrating their stable fluorescence and effective localization within the cells. Scale bar = 30 μm. Image reprinted from [75].

Figure 7 demonstrates [75] CDs emitting fluorescence within live cells, demonstrating their efficiency as cellular imaging agents. The figure highlights the uniform and stable emission within the cellular environment, affirming the potential of CDs for high-contrast, non-invasive cellular imaging applications.

For in vivo imaging, CDs show promise as they can be tailored to target tumors and specific tissues. CDs emitting in the NIR have proven especially useful for tumor imaging, as their small size and biocompatibility allow them to circulate in the bloodstream and accumulate in targeted tissues without significant immune response [70]. This makes them ideal for imaging applications where long-term tracking and high contrast are essential. CDs’ resistance to photobleaching and photoblinking further increases their reliability in providing continuous imaging over time, surpassing the performance of traditional dyes and fluorophores.

However, CDs face challenges regarding batch-to-batch consistency, as synthesis methods can yield particles with varying size distributions and emission characteristics. This inconsistency affects their imaging reliability and limits their application in clinical settings unless standardized synthesis protocols are developed. Moreover, while CDs are generally non-toxic, comprehensive long-term biocompatibility studies are needed to confirm their safety, especially in vivo, where the interaction with biological systems may vary over time [69,73].

Advantages of CDs include their high photostability, tunable fluorescence, and biocompatibility, making them suitable for both in vitro and in vivo imaging applications. Additionally, their ease of functionalization allows for targeted imaging, enhancing the specificity of bioimaging techniques. Some disadvantages involve variability in synthesis and the need for standardized protocols to ensure consistency in size and fluorescence properties. Further studies are necessary to fully understand their long-term effects within biological environments.

### 2.7. Silica Nanoparticles

Silica nanoparticles (SiO_2_ NPs) have gained prominence in bioimaging due to their versatility, biocompatibility, and stability. These NPs are typically synthesized with a core-shell structure that encapsulates fluorescent dyes or quantum dots, providing a stable fluorescence signal while preventing photobleaching over extended imaging periods. This stability is particularly beneficial for real-time cellular tracking and in vivo applications, as the silica matrix maintains the integrity of the encapsulated fluorescent material, ensuring prolonged brightness and photostability [76,77].

One of the notable properties of SiO_2_ NPs in bioimaging is their compatibility with both visible and NIR fluorescence, making them suitable for deep-tissue imaging. The transparency of silica within the biological window (650–1350 nm) minimizes light scattering, allowing SiO_2_ NPs to emit fluorescence at wavelengths optimal for biological imaging. Additionally, functionalization techniques allow for the attachment of targeting ligands, such as antibodies or peptides, enhancing specificity for certain cell types or tissue structures. This functionalization is especially advantageous for cancer imaging, as SiO_2_ NPs can accumulate preferentially in tumor tissues through the enhanced permeability and retention (EPR) effect, offering targeted imaging capabilities [56,78].

Moreover, SiO_2_ NPs serve as effective multimodal imaging agents by integrating various imaging modalities within a single particle. For example, SiO_2_ NPs can be combined with magnetic or radiopaque materials to create dual-imaging systems, enhancing diagnostic accuracy through complementary imaging techniques, such as magnetic resonance imaging (MRI) or computed tomography (CT). This versatility enables a more comprehensive approach to diagnostics and treatment monitoring, particularly in complex biological environments where different imaging modalities provide unique insights [76,78].

SiO_2_ NPs are also increasingly used in theranostics, combining imaging and therapeutic functionalities. By loading therapeutic agents onto SiO_2_ NPs, researchers can achieve simultaneous imaging and drug delivery, allowing for real-time tracking of treatment distribution within the body. This approach enhances the precision of drug delivery, reduces side effects, and allows clinicians to monitor therapeutic efficacy, making SiO_2_ NPs a promising tool for precision medicine applications [77].

## 3. Biocompatible and Biodegradable Nanoparticles for Short-Term Bioimaging

For many in vivo applications, and particularly for short-term bioimaging, biodegradability of fluorescent NPs is of importance. One example is cancer surgery, which is often used in combination with radio-, and/or chemo-, and hormonal therapy to minimize the burden of residual cancer cells of solid tumors. Currently, surgery relies on the visual appearance and palpation of the tumor, where there is a high probability that some tumor cells are missed. The complete removal of tumor cells is crucial since the presence of remaining tumor cells in the area surrounding the resection is usually considered one of the strongest predictors of tumor recurrence [79]. Therefore, there is a need to develop methods to decrease the incidence of leaving residual cancer cells behind, in particular, for tumors that are difficult to differentiate from adjacent normal tissues, such as breast cancer, and for tumors that are closed to crucial structures, such as brain tumors [79]. Optical imaging techniques are advantageous over other imaging techniques due to their amiability for real-time visualization of the tumors, facilitating intra-operative image-guided surgery. The use of fluorescently labeled markers, where tumors and nerves can be displayed in real time intra-operatively in contrasting pseudocolors, has been investigated [2].

### 3.1. Metal Oxide Nanomaterials

In the last few years, studies into metal oxide NPs have become a distinctive subject of research. Studies are undertaken into both physical implementations of these structures and their applications. This increase in interest is driven by the numerous applications of metal oxide materials in industry, medicine, information technology, energy storage, sensing, and many others [80]. The antibacterial and antithrombotic properties of metal oxides have been well explored for many pharmaceutical and biomedical applications. Individual antibacterial properties of metal oxide NPs and some combinations of them have been studied [81].

There are a large number of applications where it is essential to detect and track such fluorescent NPs without additional tags. Current methods for the detection and tracking of ZnO, TiO_2_, magnesium oxide (MgO), and other NPs in living cells use either clinical methods like blood and urine sample collections [82] or imaging via additional fluorescent markers [83]. On the other hand, intrinsic fluorescence from metal oxides has been investigated in material science and applied for many industrial applications but is rarely used in bioimaging [84]. However, the mechanism of visible and infrared fluorescence from optical defects within metal oxides, suitable for bioimaging, has been highly debated. Material scientists do not have a general consensus on the origin of these optical defects [85,86]. It has been recently found that the zinc oxide structure is efficiently excited via a nonlinear optical process of simultaneous absorption of two or three photons under illumination by an ultrashort-pulse laser that shifts the ZnO excitation band to the infrared range, suitable for bioimaging [87]. Furthermore, bright single photon emission (>650 nm) from ZnO has been shown when pumped with a laser at 532 nm [88]: all ideal properties for detection and tracking in living cells. ZnO NPs themselves can act as bright, single photon sources and can, therefore, be used as fluorescent biomarkers in their own right [89]. This discovery enables the possibility of using highly sensitive scanning confocal fluorescence techniques to track ZnO NP propagation through biological tissue at the single NP level.

**Figure 8:**
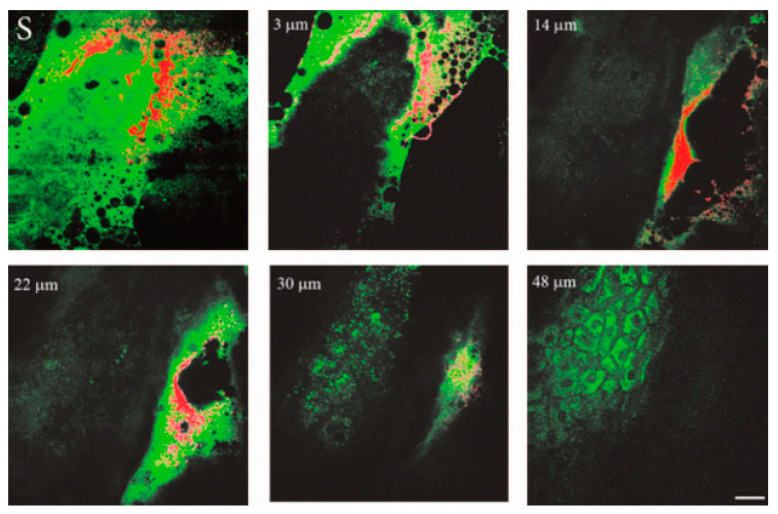
In vivo images of human skin (green) treated with ZnO commercial formulation (red). En face optical sections of the skin are displayed from top left to bottom right at depths of 0 (S), 3, 14, 22, 30, and 48 µm from the skin surface. ZnO-nano predominantly remained on the topmost layer of stratum corneum within a several-micrometer layer. No penetration of ZnO-nano into the cells or extracellular space was observed. Image reprinted from [90].

Even though ZnO NPs have been extensively used in commercial products, such as sunscreens, pharmaceutical products, and cosmetics, their toxicity is still a matter of debate. Bulk ZnO has been generally recognized as safe, but there is less certainty regarding nano-sized ZnO because of its size, which is similar to the size of biomolecules such as DNA, enzymes, and proteins present in living cells. In vitro and in vivo skin penetrability to ZnO NPs of the size range of 15 to 30 nm has been investigated using multiphoton microscopy, as shown in Figure 8 [90]. In general, toxicity depends on the concentration and physico-chemical properties of NPs, such as size, shape, composition, surface charge, and surface corona.

Currently, the research conducted into the use of MgO in biological fields has been mainly centered on its antibacterial [91] and cytotoxic [92] properties. However, certain optical defects in MgO are known to satisfy many of the requirements of a biomarker, including an absorption spectrum up to 550 nm and spectrally resolvable emission peaks above 700 nm. The material is also known to be biodegradable [93], has a long fluorescence decay time (in the order of milliseconds) [94], and is widely available. Of importance to the bioimaging applications are rarely studied defects, such as vanadium and chromium ion substitutional defects. The NIR signals from a bulk MgO sample excited by 325 nm and 532 nm radiation have been studied [95]. This study assigns the 688 nm and 700 nm peaks, only excited by 532 nm radiation, and the broad 800 nm peak to Cr^3+^ substitutional defects. Additionally, the peaks above ∼850 nm have been assigned to V^2+^ substitutional defects. It has been shown that these optical defects, attributed to vanadium and chromium ion substitutional defects, emitting in the NIR, were observed at room temperature in MgO NPs. These NPs have been successfully integrated into cultured cells, and photostable bright in vitro emission from NPs was recorded and analyzed [96].

**Figure 9:**
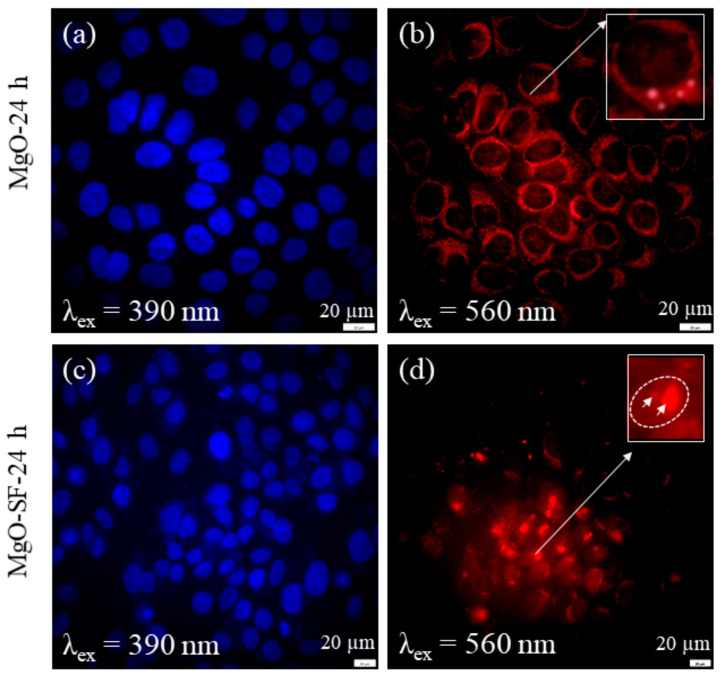
Enhanced cellular uptake and improved intracellular mobilities of MgO-SF spheres compared to MgO NPs. Wide-field fluorescence images of HaCaT cells incubated for 24 h with (**a**,**b**) MgO NPs and (**c**,**d**) MgO-SF NPs under 390 and 560 nm excitation. White box highlights the very weak fluorescence from three MgO NPs present in the cell membrane. The arrows in inset (**d**) indicate two fluorescent, bright red MgO-SF spheres. Image reprinted from [97].

However, the quantum yield and brightness of MgO NPs may be suitable for in vitro imaging, but it needs to be improved for in vivo applications. The silk fibroin-coated MgO NPs demonstrated enhanced emission efficiency compared to noncoated MgO NPs, see Figure 9 [97]. Furthermore, silk coating was found to overcome agglomeration limitations of the MgO NPs and increase in vitro mobility.

### 3.2. Synthetic Polymers

Naturally derived polymers are generally biodegradable, but they usually have an uncontrolled rate of degradation. On the other hand, synthetic polymers exhibit good controllability in terms of degradation rate and behavior, but they generally lack inherent biological activity. There is a range of biodegradable polymers that provide good control of the rate of degradation, and they are usually used for drug delivery [13]. In terms of fluorescence, polymers can be distinguished into two major types: (1) polymer-conjugated FNPs are produced through the embedding of external fluorophores in a polymer matrix [98], and (2) aggregation-induced emission (AIE)-based polymers [99]. In the first case, both materials, polymer matrix and fluorescent probe, would need to be biocompatible and biodegradable to satisfy requirements for short-term bioimaging. However, fluorescent polymers with aromatic repeating units are not desirable in biomedical applications as they are typically hydrophobic, nonbiodegradable, and potentially toxic. A series of non-conventional biogenic and synthetic polymers have been reported, showing fluoresce in the condensed phase [100].

The importance of AIE-induced fluorescence is demonstrated in nonfluorescent polymers, mainly for sensing and biological applications [101]. This phenomenon was initially reported in 2001, demonstrating very strong luminescence at 492 nm, excited at 381 nm, from the nanoaggregates of 1-methyl1,2,3,4,5-pentaphenylsilole in an EtOH/H_2_O medium or in the solid state [102]. Over the past few decades, polymeric nanomedicines have become increasingly important for drug delivery. Progress in the field of bioimaging is rapidly creating new opportunities for polymeric nanomedicine to advance bioimaging systems. To realize the clinical potential of such sophisticated bioimaging probes, effective in vivo delivery technologies are obviously required [103].

### 3.3. Dendrimers, Lipids, and Micelles

These NPs have been used as drug carriers in nanomedicine, enabling slow and controlled release and targeted drug delivery [13].

Dendrimers are small NPs, with sizes in the one-digit nm scale. Their brightness can be controlled by size and color by the terminal fluorophore. They have been applied to plain imaging, targeted imaging, and sensing/imaging in combination with some fluorophores, like dyes and QDs [104]. However, in this review, we only point out the intrinsic fluorescence of dendrimers, usually referred to as non-traditional intrinsic fluorescence (NTIF) [105,106]. The origin of intrinsic fluorescence has not yet been fully understood, and yet there are already reports on the use of intrinsically fluorescent dendrimers for bioimaging [107], where intrinsically fluorescent dendrimers of different size and charge were used as nanoprobes of cell transport without the need for fluorescent labeling. However, for future bioimaging applications, it is essential to enhance their autofluorescence without using additional agents.

Lipid-based NPs, such as liposomes (LPs) and lipid nanoparticles (LNPs), have been the subject of substantial research as potential carriers of active compounds often used in drug delivery [108]. If these NPs are used in bioimaging, they are usually loaded with fluorescent probes. For example, Gravier et al. loaded lipid NPs with fluorophores (NIR dyes, including Indocyanine Green) for use in multichannel in vivo imaging of lymph nodes in mice [109]. However, in order to be fully biocompatible, lipid-based NPs should encapsulate either biocompatible and biodegradable fluorophores or intrinsic fluorescence of lipids used. Indeed, lipids exhibit bright fluorescence in red, as it has been shown that lipids are present and exhibit bright red fluorescence in parasitic egg membranes [110]. Lipid fluorescence has been demonstrated in vivo, contributing to the overall emission signal of the liver tissue [111]. Moreover, according to Chen et al., the lipid droplets can act as endogenous intracellular microlenses that open the intriguing route for a multifunctional biocompatible optics tool for biosensing, endoscopic imaging, and single-cell diagnosis [112].

Various fluorescent tags can be incorporated with micelles to create efficient fluorescent probes that can be utilized for sensing and bioimaging [113,114]. However, in this review, we point out the intrinsic fluorescence of these nanomaterials.

**Figure 10:**
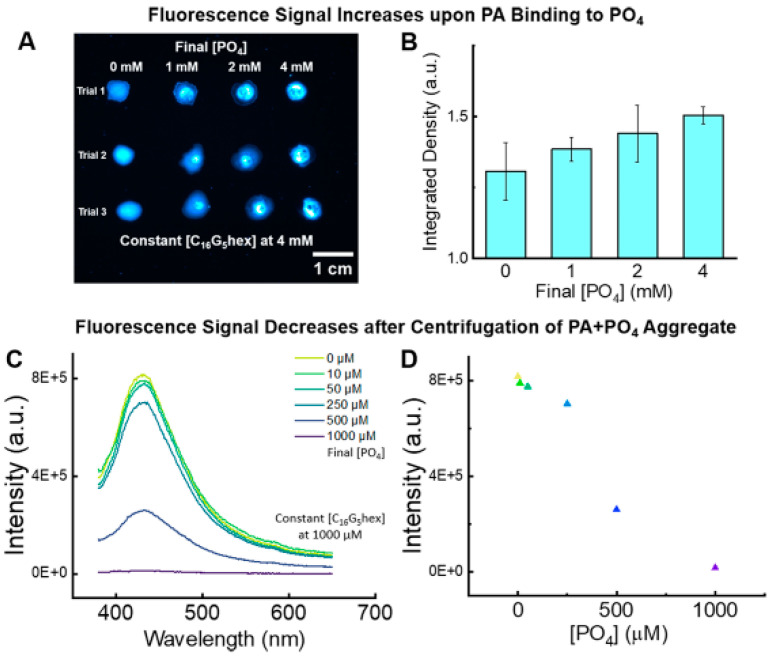
Application of peptide amphiphile micelles (PAMs) intrinsic fluorescence to protein-inspired phosphate sensing. (**A**) Fluorescence gel microscopy images of PAM droplets with increasing final concentration of phosphate. (**B**) Integrated fluorescence density of droplet images averaged across three trials quantifies the increase in fluorescence intensity with increasing amounts of phosphate. (**C**) Emission spectra of the supernatant after centrifugation of 1000 μM PAMs with increasing amounts of phosphate. Concentration ranges from 0 µM (yellow) to 1000 µM (purple)As phosphate complexes increase with droplets, the emission decreases. (**D**) Fluorescence intensity of the peak maximums at 430 nm for each phosphate concentration, which linearly decreases as the added phosphate increases. Image reprinted from [115].

A recent paper by Whitney C. Fowler [115] has demonstrated the intrinsic fluorescence of Peptide Amphiphile Micelles and their application to protein-inspired phosphate sensing, see Figure 10. As pointed out by the author PAMs, intrinsic fluorescence becomes another highly useful feature to add to this well-studied material platform that features precise synthetic control, tunable self-assembly, and straightforward functionalization.

## 4. Summary and Perspective

Fluorescence techniques have been extensively used in biomedicine. In comparison with other methods, they are straightforward and non-invasive and have a real-time response that is important for biomedical use. In this mini-review, we have tried to bring out new candidates for short and long-term bioimaging. We have given a few examples of emerging nanomaterials for both categories. We consider that intrinsic fluorescence-based NPs are advantageous compared to NPs with extrinsic fluorophores due to minimal sample handling and no chemical processing or modification. However, all FNPs have their advantages and some drawbacks. There is certainly not an ideal type of FNP that can satisfy all requirements for different applications. Depending on the kind of application, different kinds of criteria may apply when selecting NPs. We have mainly pointed out applications in plain fluorescence imaging where brightness, no-toxicity, and inertness to their microenvironment are of importance. Note that NPs that work in the optical window longer than 600 nm in range are preferred, while probes with UV excitation should be avoided, as discussed in the Introduction. However, for certain applications like targeted or deep-tissue imaging, additional surface functionalization can enhance specificity and fluorescence stability. For instance, NIR-emitting FNPs, such as modified carbon dots and silicon carbide, facilitate greater tissue penetration and minimize background autofluorescence, making them suitable for in vivo studies. Some NPs are easier to functionalize, such as SiO_2_ and polymer NPs, whereas there are some challenges with ND functionalization.

It is a general rule that non-toxic NPs should be used for any bioimaging applications, even when they are used as carriers for toxic drug delivery. As mentioned in the Introduction, the discussion on the potential toxicity of NPs is an ongoing issue, and numerous studies have been performed to investigate the potentially harmful effects, especially for in vivo imaging. Another issue that has not been fully addressed is the environmental sustainability of these nanomaterials, which should include both fabrication and disposal of these materials in the environment. This is an evolving subject, and many green methods of fabrication have been reported in the literature [116,117,118], as well as some concerning reports on environmental pollution [119]. NPs such as gold and silver have unique imaging properties but require careful consideration due to potential cytotoxicity. In contrast, biodegradable options like metal oxides and biopolymer-based particles offer more favorable degradation profiles, aligning with green chemistry initiatives and reducing environmental impact.

Undoubtedly, more research is needed to develop aspects of NPs and their optimization. For example, the origin of intrinsic fluorescence has not yet been fully understood in dendrimers. Also, the ongoing research and development in the biomedical field demands single multifunctional composite materials that can be employed simultaneously for drug delivery and biomedical imaging. Future research must prioritize the standardization of synthesis methods to achieve consistent, safe, and eco-friendly FNPs, given the demand for sustainable practices in nanomedicine.

The diversity of FNPs reviewed here underscores that no single NP type can meet all imaging demands. Certainly, there are many more evolving nanomaterials discussed here that may satisfy requirements for non-invasive bioimaging. Indeed, all materials have the natural capability to fluoresce due to the presence of endogenous fluorophores.

## 5. Conclusions

We have discussed the potential of some relatively new biocompatible NPs for real-time and non-invasive bioimaging. First, biocompatible, chemically inert fluorescent NPs for long-term imaging have been summarized. Then, a special class of biocompatible and biodegradable NPs for short-term bioimaging has been discussed.

In conclusion, fluorescent NPs have great potential in the field of biomedical optics and could bring tremendous progress in biomedicine, but many significant challenges remain in advancing FNPs for broader clinical use. These obstacles include variations in formulations and the in vivo stability of NPs, as well as limited data on the fate and toxicity of NPs in vivo. The full potential of these NPs is yet to be realized and employed for clinical use. Environmental sustainability also emerges as a critical focus area, with green synthesis and eco-friendly disposal methods necessary as FNPs move from research to application.
